# Wolff-Parkinson-White Syndrome: A Master of Disguise

**DOI:** 10.7759/cureus.8672

**Published:** 2020-06-17

**Authors:** Amit Sapra, Janet Albers, Priyanka Bhandari, Dean Davis, Eukesh Ranjit

**Affiliations:** 1 Family Medicine, Southern Illinois University School of Medicine, Springfield, USA; 2 Family Medicine, Decatur Memorial Hospital, Decatur, USA

**Keywords:** anxiety, panic attacks, panic disorder, chest pain, chest discomfort, cardiac arrhyrthmias, palpitations, somatic symptoms of anxiety disorders, skipped beats, elusive medical condition

## Abstract

Wolff-Parkinson-White syndrome is the most common form of ventricular preexcitation and affects 1-3 per 1,000 persons worldwide. Many patients remain asymptomatic throughout their lives; however, approximately half of the patients with Wolff-Parkinson-White syndrome experience symptoms secondary to tachyarrhythmias, such as paroxysmal supraventricular tachycardia, atrial fibrillation, atrial flutter, and, rarely, ventricular fibrillation and sudden death. Patients with Wolff-Parkinson-White syndrome may present with a multitude of symptoms such as unexplained anxiety, palpitations, fatigue, light-headedness or dizziness, loss of consciousness, and shortness of breath. We report the case of a patient who presented with a plethora of symptoms related to generalized anxiety along with several confounding factors such as psychosocial stressors, chronic fatigue secondary to high physical and mental demands at work, a strong family history of anxiety, and a history of substance abuse. Keeping cardiac dysrhythmia within his differential diagnosis allowed for accurate diagnosis and treatment.

## Introduction

Wolff-Parkinson-White syndrome is characterized by an accessory pathway (bypass tract) between the atria and ventricles that conducts in parallel with the atrioventricular (AV) node-His bundle, but faster [1].

Historically, Louis Wolff, John Parkinson, and Paul D. White first described the condition in 1930 in a series of 11 healthy young people with a functional bundle branch block, an abnormally short PR interval, and paroxysms of tachycardia or atrial fibrillation (described as "auricular fibrillation") [2].

The patient who was evaluated by us presented with a very subtle plethora of symptoms that could easily be dismissed as generalized anxiety or stress secondary to his demanding work and family responsibilities. The symptoms were, in fact, a manifestation of a somewhat rare cardiac disorder requiring further workup and management. The patient's vitals and cardiovascular examination were entirely within the normal limits. A high index of suspicion was required to diagnose Wolff-Parkinson-White syndrome, which can potentially have an initial presentation of sudden cardiac death [3].

## Case presentation

A 38-year-old Caucasian male presented to the clinic with concerns of brief ongoing episodes of anxiety every couple of weeks. He described a 10-year history of episodes characterized as a squeezing sensation in the chest followed by palpitations.

He reported having numerous stressful events in the recent and remote past, and a family history significant for generalized anxiety in a number of first-degree relatives. He reported worry and concern regarding his past IV drug use possibly affecting his health at this stage in life. He also described experiencing a "sharp chest pain" that did not radiate and resolved spontaneously within a couple of minutes. These symptoms caused him to stop what he was doing at work and resulted in interference with his daily activities.

He is employed in a slaughterhouse, and he described his job as "physically and mentally demanding" as he handles live animals. He reported work exhaustion and thought stress could be contributing to his symptoms.

He denied presyncope or syncope. He also denied dyspnea on exertion or at rest. He denied any symptoms suggestive of orthopnea, paroxysmal nocturnal dyspnea, weight gain, or pedal edema.

At the time of the presentation, he had been using nicotine patches for tobacco cessation. He was not on any prescription, over-the-counter medications, supplements, or herbal remedies. Otherwise, he had no other acute health concerns.

The patient denied any family history of sudden cardiac death or cardiac arrhythmias. The patient has an extensive family history of coronary artery disease (CAD) after 50 years of age. Recently his mother was diagnosed with CAD and underwent percutaneous intervention with stent placement.

Additionally, he has a history of hepatitis C, the treatment of which concluded in 2019, and a history of intravenous drug use in sustained recovery for five years. He is a current smoker and smokes half a pack a day.

The patient's vitals and physical examination including cardiovascular examination were completely normal.

**Figure 1:**
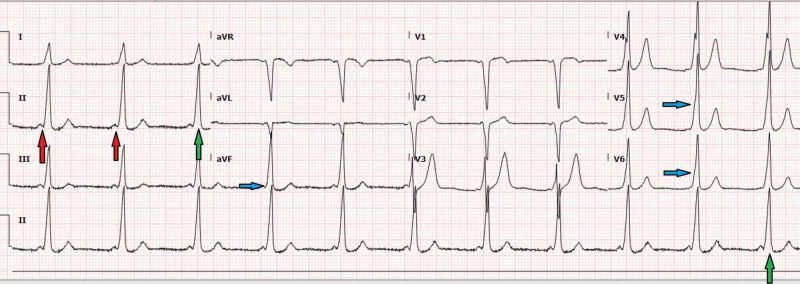
EKG ordered in the clinic showed shortened PR interval (red arrows), delta waves (blue arrows), and wide QRS complexes (green arrows). EKG, electrocardiogram

Labs were ordered in the clinic, which included complete blood count to rule out anemia, complete metabolic profile to rule out electrolyte abnormalities, and lipid profile to assess the risk of cardiac disease. Thyroid-stimulating hormone was obtained to evaluate palpitations. Chantix® (varenicline) starter pack was prescribed to help him quit smoking. An electrocardiogram (EKG) was ordered to assess chest discomfort (Figure 1).

**Figure 2:**
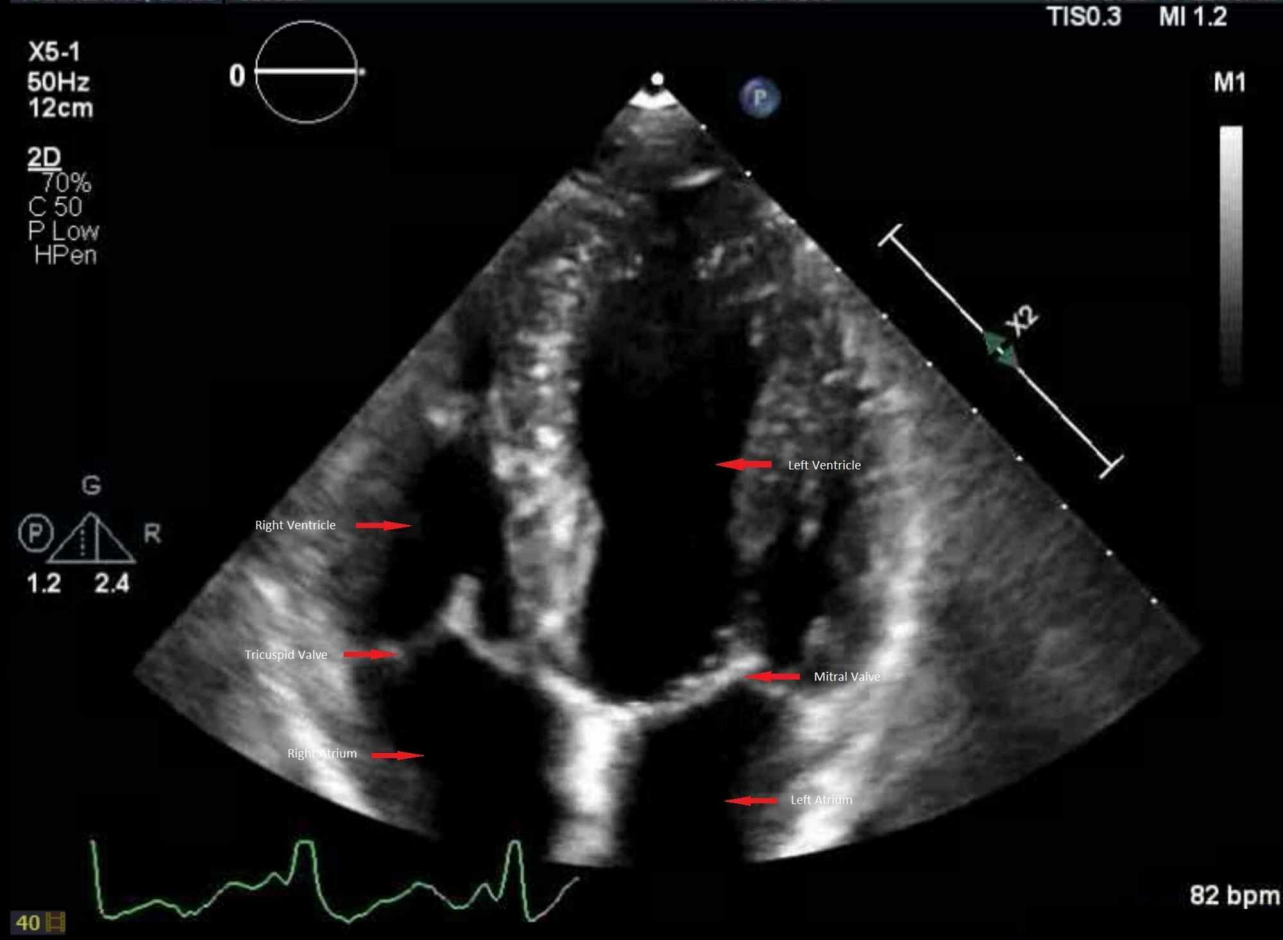
Echocardiogram of the patient with labelled chambers and valves (red arrows), which was found to be within the normal limits.

An echocardiogram was ordered to assess valvular structure given his history of palpitations and IV drug use, and it revealed an ejection fraction of 63% with normal right and left ventricular function and no valvular abnormalities (Figure 2).

The patient’s labs were within normal limits. EKG revealed Wolff-Parkinson-White syndrome. Chantix was held due to the EKG findings.

The patient was urgently referred to the electrophysiology clinic, and after evaluation by the electrophysiology cardiologist, he underwent catheter ablation. During the procedure, it was found that he had two pathways, one anterolateral right and the other Para-Hisian, which has a high risk of complete heart block. The procedure was hence aborted, and the patient was advised to undergo cryoablation.

## Discussion

Wolff-Parkinson-White syndrome is the most common form of ventricular preexcitation [4]. It is a conduction disorder of the heart caused by a pre-excitation accessory pathway resulting in tachyarrhythmias [5]. The definition of Wolff-Parkinson-White syndrome relies on the following electrocardiographic features: (1) a PR interval less than 0.12 seconds, (2) a slurring of the initial segment of the QRS complex, known as a delta wave, (3) a QRS complex widening with a total duration greater than 0.12 seconds, and (4) secondary repolarization changes reflected in ST segment-T wave changes that are generally directed opposite (discordant) to the major delta wave and QRS complex changes [6].

Additionally, Wolff-Parkinson-White syndrome is differentiated from Wolff-Parkinson-White pattern on EKG recordings, as these patients are asymptomatic. Wolff-Parkinson-White syndrome affects 1-3 per 1,000 persons worldwide and is the most prevalent cause of arrhythmia in Chinese persons [7]. Although the vast majority of Wolff-Parkinson-White syndrome cases have an unknown etiology, a small but unclear percentage of cases are associated with the *PRKAG2* gene [7]. A formal review of the etiology of Wolff-Parkinson-White syndrome is beyond the focus of this article.

Patients with Wolff-Parkinson-White syndrome may present with a multitude of symptoms such as unexplained anxiety, palpitations, fatigue, light-headedness or dizziness, loss of consciousness, and shortness of breath [8].

Even though many patients remain asymptomatic throughout their lives, approximately half of the patients with Wolff-Parkinson-White syndrome experience symptoms secondary to tachyarrhythmias, such as atrial fibrillation, atrial flutter, paroxysmal supraventricular tachycardia, and, rarely, ventricular fibrillation and sudden death [4]. The overall risk of sudden cardiac death in Wolff-Parkinson-White syndrome is estimated at 0.1% per year in asymptomatic patients and 0.3% per year in symptomatic patients. Ventricular fibrillation is generally secondary to atrial fibrillation, leading to an extremely rapid ventricular response in the presence of an accessory pathway with a critically short anterograde refractory period (usually <250 ms). Shortened refractory period, and the accessory pathway, overrides the default AV nodal-His-Purkinje pathway. Therefore, electrical impulses are transmitted through the accessory pathway more readily, leading to the aforementioned arrhythmias [9]. Sudden death can occur even in completely asymptomatic patients; therefore, this risk should be carefully considered any time Wolff-Parkinson-White syndrome is diagnosed [10].

This patient presented with feelings of anxiety accompanied by psychosocial stressors, chronic fatigue secondary to high physical and mental demands at work, a strong family history of anxiety, and a history of substance abuse. Keeping cardiac dysrhythmia within his differential diagnosis allowed for accurate diagnosis and treatment.

After diagnosis, the patient joined social media support groups for Wolff-Parkinson-White syndrome and soon realized that he was not alone in his anxiety presentation.

## Conclusions

This case report invites us to remain critical and vigilant when assessing patients with anxiety symptoms that may be considered “psychosomatic”. Less common disorders such as Wolff-Parkinson-White syndrome can easily be overlooked. Our patient presented with symptoms suggestive of generalized anxiety along with chronic fatigue due to multiple psychosocial factors. Keeping cardiac dysrhythmia within his differential diagnosis allowed for accurate diagnosis and treatment. He and many others within his support group have expressed similar presentations of anxiety. According to the patient, many members of his Wolff-Parkinson-White support group went undiagnosed for months to years while undergoing psychotherapy and medical management for anxiety disorders and were eventually diagnosed with Wolff-Parkinson-White syndrome. More research needs to be conducted to further study these anecdotal claims. While evaluating patients for anxiety, primary care providers must keep a broad differential diagnosis in mind.
